# Supplementary material for: Comparison of plasma soluble and extracellular vesicles-associated biomarkers in Alzheimer’s disease patients and cognitively normal individuals
Source: Alzheimers Res Ther. 2024 Jun 28;16:141. doi: 10.1186/s13195-024-01508-6 (PMC11212434; doi:10.1186/s13195-024-01508-6)
Supplement: Supplementary file 1 — Supplementary Material 1 [file 13195_2024_1508_MOESM1_ESM.docx]

## **
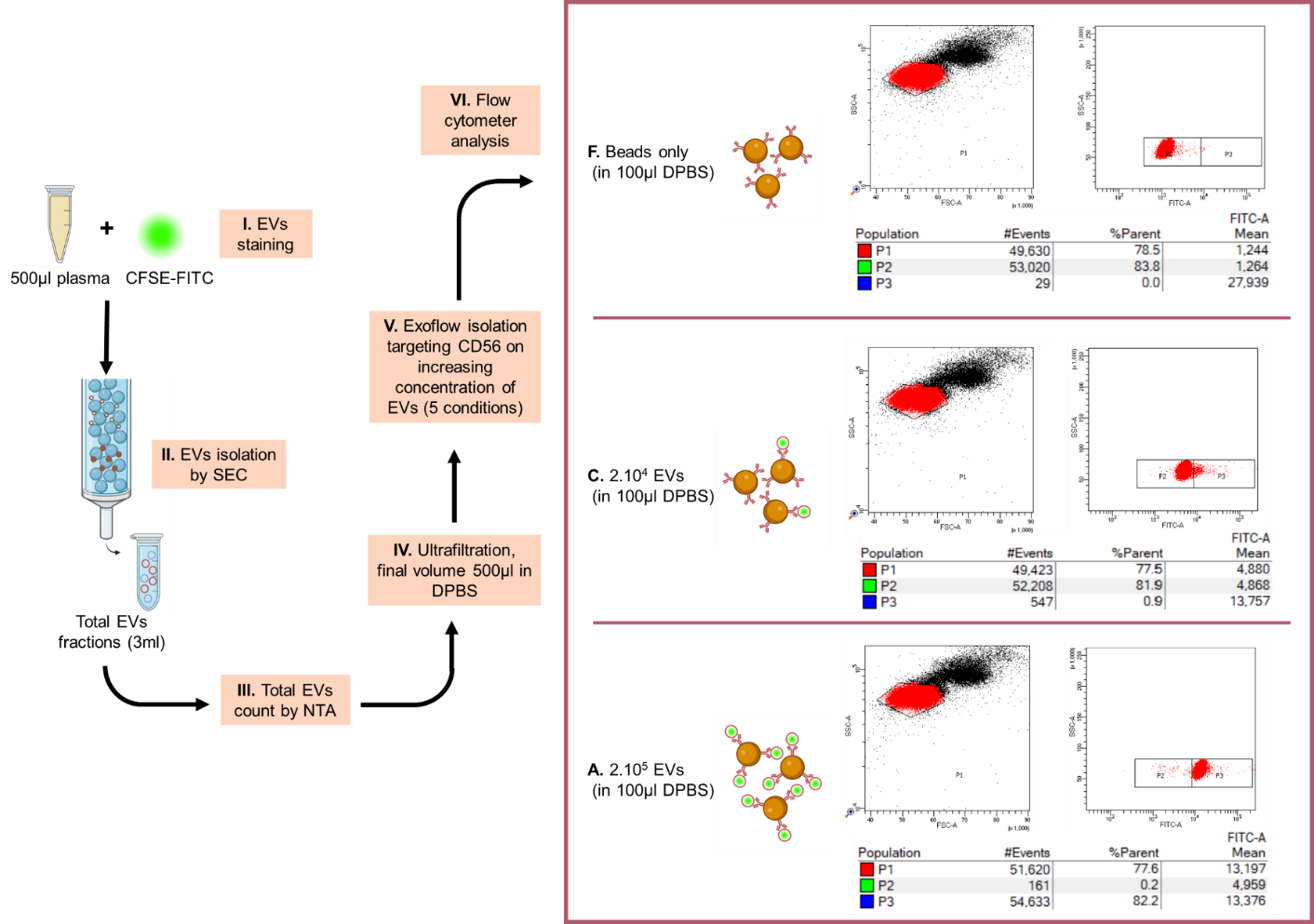
Supplementary files**

**Supplementary file 1: Validation protocol for NDEVs isolation using Exoflow technique.**

**I.** Plasma sample was incubated with 6µl of CFSE-FITC at RT for 2h. **II**. EVs were isolated by size exclusion chromatography, by collecting EVS fractions (F7 to 12 = 3ml total volume). **III.** Number of EVs were count by NTA to determine EVs quantity to use in Exolfow protocol. **IV.** 3ml of eluted EVs were concentrated by ultrafiltration (10kDa filter, 4°C, 45min at 4000g), final volume was 500µl. **V.** Exoflow with anti-CD56 antibody was performed in 5 different conditions : A. 100µl of concentrated EVs (+/–2.10^5^ EVs), B.50µl (+/– 1.10^5^ EVs), C.10µl (+/– 2.10^4^ EVs), D.5µl (+/– 1.10^4^ EVs), E.1µl (+/– 2.10^3^ EVs) and F. magnetic beads without EVs. We used the same amount of beads solution (40µl) and antibody (10µl, dilution 1/5). Exoflow isolation was performed following manufacturer protocol (System Biosciences, #CSFLOWBASICA-1). **VI.** Post isolation beads were analyzed by flow cytometry for FITC signal. Beads population was selected based on size scatter and forward scatter (population P1), and with negative FITC signal (population P2). Positive population (P3) was considered positive above log10^4^ of FITC signal. Saturation point was reach with condition A. 2.10^5^ EVs.

**
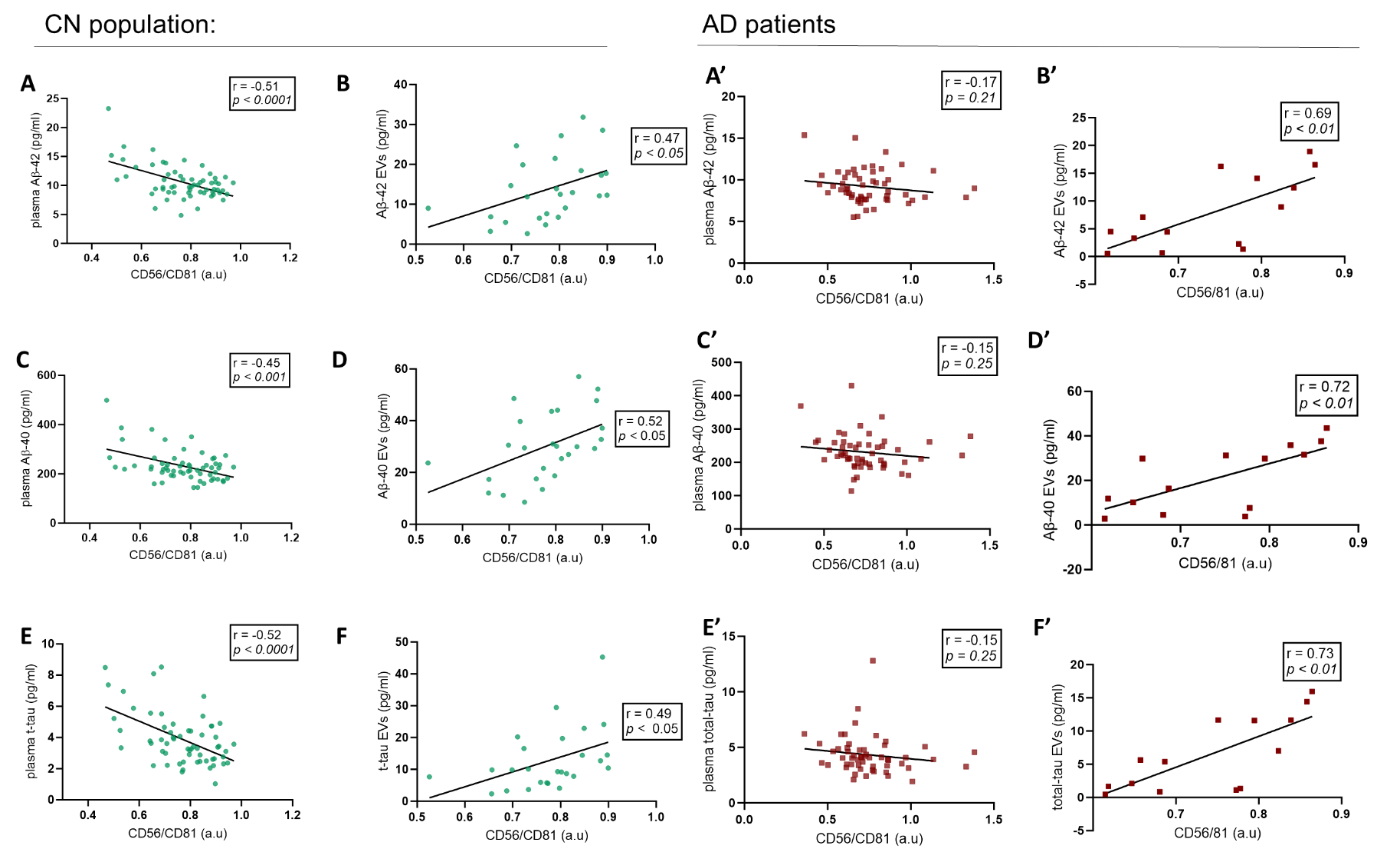
**

**Supplementary file 2: Detailed scatter plots of the correlation between plasma soluble biomarkers quantity, plasma NDEVs quantity and their content in AD biomarkers.**

Population details for figures A, A’, C, C’, E and E’ can be found on paragraph 3.2. For the analysis of NDEVs content, we focused on subgroups distributed across the plasma NDEVs spectrum, ranging from low circulating participants to high circulating participants. In the CN group, n=27, with a mean MMSE [SD] of 28.76 [1.27], mean age [SD] of 61.78 [14.8], 16 women [59.3%], and mean CD56/81 of 0.768 [0.10]. In the AD group, n=14, with a mean MMSE [SD] of 21.9 [5.09], mean age [SD] of 66.4 [16.7], 6 women [42.8%], and mean CD56/81 of 0.742 [0.09].


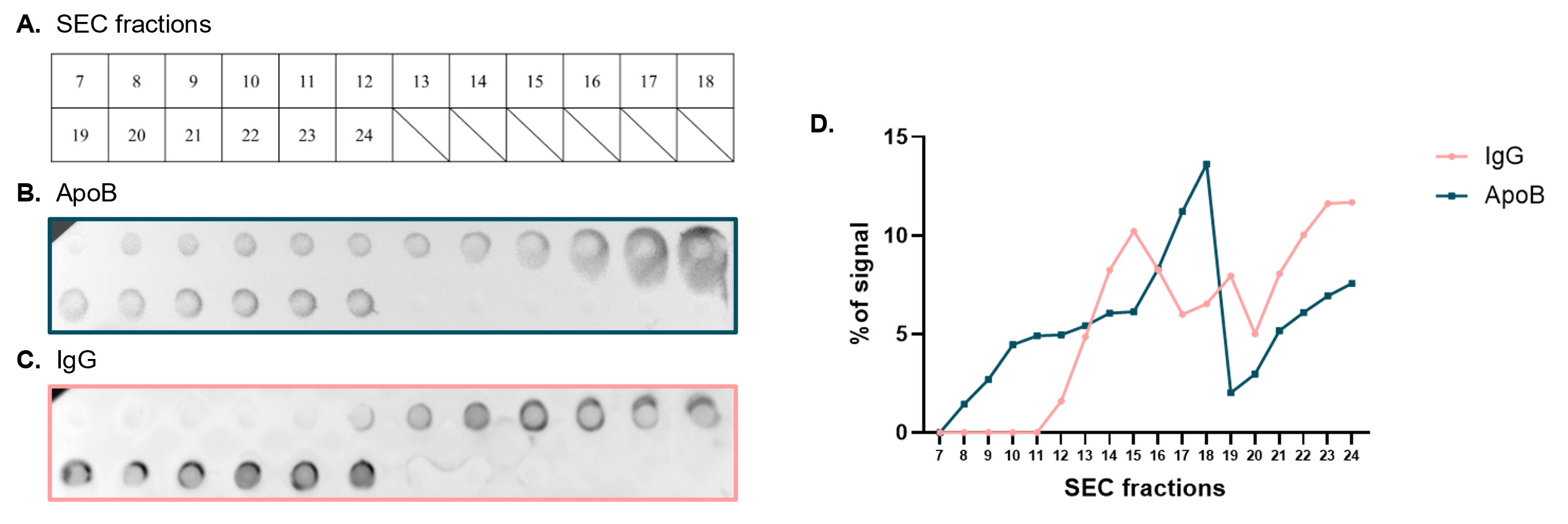
**Supplementary file 3: Dot blot results for EVs characterization**

*Exclusion markers tested are apolipoprotein B (ApoB) and human IgG.***A.** Schematic display of the dot blots showing the localization of the fractions spotted on the membrane (fractions 7 to 24). Fractions before 7 were not analyzed due to the absence of particles detected by NTA in fractions 1 to 6. Putative EV particles were present in fractions 7 to 12. **B.** Raw dot blot results for ApoB detection (protocol detail in section 2.6.3). **C.** Raw dot blot result for IgG detection. **D.** Quantification of the signal in dot blots for ApoB and IgG. IgG is detected mostly after fraction 12 (98.41% of the signal), while the ApoB signal is detected in fractions 7 to 12 (18.46% of the signal), with the signal peaking in fractions 13 to 24 (81.54% of the signal).
